# Multi-site medical record review for validation of intentional self-harm coding in emergency departments

**DOI:** 10.1186/s40621-022-00380-y

**Published:** 2022-06-07

**Authors:** Barbara A. Gabella, Beth Hume, Linda Li, Marianne Mabida, Julia Costich

**Affiliations:** 1 Colorado Department of Public Health and Environment, 4300 Cherry Creek Drive South, A4, Denver, CO 80246-1530 USA; 2 Massachusetts Department of Public Health, Boston, MA USA; 3 Maryland Department of Health, Baltimore, MD USA; 4 Department of Health Management and Policy and Kentucky Injury Prevention and Research Center, University of Kentucky College of Public Health, Lexington, KY USA

**Keywords:** Intentional self-harm, Suicide, Population surveillance, ICD-10-CM

## Abstract

**Background:**

Codes in the International Classification of Diseases, Tenth Revision, Clinical Modification (ICD-10-CM), are used for injury surveillance, including surveillance of intentional self-harm, as they appear in administrative billing records. This study estimated the positive predictive value of ICD-10-CM codes for intentional self-harm in emergency department (ED) billing records for patients aged 10 years and older who did not die and were not admitted to an inpatient medical service.

**Methods:**

The study team in Maryland, Colorado, and Massachusetts selected all or a random sample of ED billing records with an ICD-10-CM code for intentional self-harm (specific codes that began with X71-X83, T36-T65, T71, T14.91). Positive predictive value (PPV) was determined by the number and percentage of records with a physician diagnosis of intentional self-harm, based on a retrospective review of the original medical record.

**Results:**

The estimated PPV for the codes’ capture of intentional self-harm based on physician diagnosis in the original medical record was 89.8% (95% CI 85.0–93.4) for Maryland records, 91.9% (95% CI 87.7–95.0) for Colorado records, and 97.3% (95% CI 95.1–98.7) for Massachusetts records.

**Conclusion:**

Given the high PPV of the codes, epidemiologists can use the codes for public health surveillance of intentional self-harm treated in the ED using ICD-10-CM coded administrative billing records. However, these codes and related variables in the billing database cannot definitively distinguish between suicidal and non-suicidal intentional self-harm.

**Supplementary Information:**

The online version contains supplementary material available at 10.1186/s40621-022-00380-y.

## Background

Effective injury prevention requires timely and accurate data on causes of injury morbidity and mortality (Data and Surveillance Task Force 2014; Injury Surveillance Workgroup 9 2016), including intentional self-harm. A type of intentional self-harm, suicide, is an increasingly common cause of death (Hedegaard et al. 2021), but quantifying suicide attempts and related diagnoses poses many challenges (Walkup et al. 2012; Swain et al. 2019; Hansen et al. 2021). These challenges reflect the continuum of suicidality, reliance on patients’ articulation of their intent to die, and the often-transitory nature of the crisis period (Posner et al. 2007). Stigma is also a factor in patients’ under-reporting of suicidal intent and under-coding of both suicide attempts and deaths by suicide (Frey et al. 2016; Corrigan et al. 2017). These challenges are particularly salient for patients seen in hospital emergency departments (EDs) because of the relatively short length of their visits.

Intentional self-harm also includes purposeful self-harm but without the intent to die. This behavior may or may not lead to injury that requires medical treatment. Mild forms of non-suicidal intentional self-harm include scratching or plucking hair. Potentially more severe forms include cutting, burning, or hitting oneself (Halicka and Kiejna 2018; Taylor et al. 2018, 2019). Documented functions of this behavior include managing distress or regulating emotion, providing a sense of mastery, punishment, or exerting interpersonal influence (Emondson et al. 2016; Taylor et al. 2018, 2019) and can be characterized as positive or negative reinforcement (Halicka and Kiejna 2018) or intrapersonal and interpersonal (Taylor et al. 2018, 2019).

Codes in the clinical modification of International Classification of Diseases have long been used for injury surveillance as they appear in administrative billing records (Swain et al. 2019; Walkup et al. 2012; Hedegaard et al. 2018). On October 1, 2015, the USA implemented the ICD-10-CM system. Like previous clinical modifications, its primary purpose was to support accurate health care billing and reimbursement rather than epidemiological surveillance. In the USA, coding rules are based on key terms and documentation from the clinician in whose name the billing records are submitted to third-party payers. As with other data sources used for public health surveillance, “because the information is collected for other purposes, the use of standardized case definitions and the quality of the data collected can be challenging” (Data and Surveillance Task Force 2014).

ICD-10-CM has expanded the range of injury-related codes, including diagnostic and external cause codes for intentional self-harm (Hedegaard et al. 2018). The expansion did not address that codes and coding guidelines obscure the distinction between self-harm with intent to die (suicide attempt) and self-harm not intended to be lethal. The narrative label “intentional self-harm” used in ICD-10-CM is a change from “suicide and self-inflicted injury” in ICD-9-CM (Hedegaard et al. 2018). The only label for an ICD-10-CM code that uses the terminology “suicide attempt” is T14.91, although its use is appropriate only when the specific means of the suicide attempt and the nature of injury is unknown, according to ICD-10-CM guidelines (Hedegaard et al. 2018). Although versions of ICD-10 have been adopted outside of the USA since the 1990s, the international literature specific to coding of self-harm visits is sparse (Callahan et al. 2013; Randall et al. 2017; Sveticic et al. 2020). The impact of the US ICD-10-CM on intentional self-harm surveillance and suicide attempt surveillance remains unclear.

The main objective of this study is to estimate the positive predictive value (PPV) of the ICD-10-CM codes for intentional self-harm in ED billing records for patients aged 10 years and older, that is, to estimate the proportion of billing records with physician documentation of intentional self-harm in the corresponding medical record. A secondary objective is to determine whether ICD-10-CM coding adequately distinguishes between suicide attempts and non-suicidal intentional self-harm in this group.

## Methods

The study design was a retrospective, cross-sectional review of medical records at emergency departments in the states of Colorado and Massachusetts as well as one large hospital emergency department in Maryland. To assess positive predictive value for intentional self-harm, each study site team selected all or a random sample of ED billing records based on any ICD-10-CM code for intentional self-harm (Hedegaard et al. 2018). The study setting was EDs affiliated with non-federal acute care hospitals. The dates, number of hospitals, and size of the samples varied, as shown in Table 1.

**Table 1:** Setting and other study characteristics: medical record review of intentional self-harm treated in EDs

	Maryland	Colorado	Massachusetts
	Large academic hospital	All acute care hospitals in the state	All acute care hospitals in the state
Number of hospital EDs in the study (in the sampling frame)	1	80^a^	71
Number of records in random sample	234	260	400
% of state population covered by hospital(s) (catchment area)	15%	100%	100%
Dates of the ED visits in the billing data set	Jan 2018–Dec 2019	Jan 2019–Dec 2019	Oct 2017–Sep 2018
Number of diagnosis fields in the ED billing data set	32	30 + 3 cause fields	30 + 1 cause field
Medical record reviewer	10 clinical researchers	1 professional coder	3 professional coders
Data source	Electronic medical record	ED report and EMS transport^b^	Full ER record^c^

*ED* emergency department, *EMS* emergency medical services, *ER* emergency room

^a^80 of 81 reported in 2019. The ED visits at the non-reporting hospital with two ED locations accounted for less than 0.001% of all Colorado ED visits in 2019

^b^Also requested: face sheet, triage reports, toxicology and laboratory reports, history and physical, social worker notes, physician notes, psychiatry notes, consultations, discharge summary, and ICD-10-CM codes

^c^Full ER record including nursing and physician progress notes, EMS reports, behavioral and psychiatric consults, and social service notes

### ICD-10-CM codes for intentional self-harm

The study focused on ICD-10-CM codes for intentional self-harm coded in emergency department discharge records, grouped in Hedegaard et al. (2018) as follows:

1. Drowning/submersion, firearm, explosive material, fire/flame, hot vapors/objects, sharp object, blunt object, jumping from a high place, jumping or lying in front of a moving object, crashing of motor vehicle, or other specified means: X71–X83
2. Poisoning by drugs, medications, and biological substances: T36–T50 with the 6th character of the ICD-10-CM code of “2,” except for T36.9, T37.9, T39.9, T41.4, T42.7, T43.9, T45.9, T47.9, and T49.9, which are included if the 5th character of the code is “2”
3. Toxic effects of non-medicinal substances: T51–T65 with the 6th character of the ICD-10-CM code of “2,” except for T51.9, T52.9, T53.9, T54.9, T56.9, T57.9, T58.0, T58.1, T58.9, T59.9, T60.9, T61.0, T61.1, T61.9, T62.9, T63.9, T64.0, T64.8, and T65.9, which are included if the 5th character of the code is “2”
4. Asphyxiation, suffocation, hanging: T71 with the 6th character of the ICD-10-CM code of “2”
5. Suicide attempt: T14.91

### Study population and data sources

The study population was persons aged 10 and older treated in emergency departments for intentional self-harm as indicated by the diagnosis codes on the ED billing records. The study used two data sources: (1) statewide administrative billing records for ED visits to select records eligible for this study and (2) the corresponding original medical record to collect physician diagnoses and documented intent, injury, and patient characteristics. Eligible records were ED billing records with (1) an ICD-10-CM code for intentional self-harm in any of their diagnosis fields (for the codes beginning with T) or external cause fields, if separate fields (for the codes beginning with X), (2) patient aged 10 years and older, and (3) discharge disposition indicating that the patient did not die and was not admitted for inpatient medical care at the affiliated acute care hospital. Including ages 10–17 years old ensured that the results apply to a broad indicator of intentional self-harm. Excluding patients who died or were admitted to the hospital inpatient medical service made this sample comparable to national injury surveillance methods using administrative billing records (Centers for Disease Control and Prevention 2021). Not all patients sustained injuries needing medical treatment. The patients could have been discharged home or to a psychiatric unit in the same or another hospital. The sites varied in lag time for receiving ED billing data and time to accumulate an adequate sample size of eligible ED records, resulting in different study periods for each site (Table 1). These methods are similar to those reported in assessments of ICD-10-CM code sets for other injuries (Gabella et al. 2021; Hansen et al. 2021; Peterson et al. 2021).

### Variables of interest from medical records

To reduce systematic differences (observer bias) among the reviewers and over time, the study team developed a common abstraction form to record the variables of interest from the original medical record and a detailed reviewer manual with definitions and examples, including clinical terms and explanation of ICD-10-CM coding rules. Medical record reviewers were clinical researchers at one site and professional medical coders at other sites (Table 1). These reviewers assessed physician documentation for self-harm keywords that support the assignment of any intentional self-harm ICD-10-CM code as well as the type of self-harm intent (suicidal or non-suicidal). The reviewers abstracted the mechanism of injury: cut or pierce; poisoning by drugs, medication, or biological substance; toxic effects of non-medicinal substance; suffocation, asphyxiation, hanging, strangulation; and other (firearm, drowning or submersion, jumping or intentional fall, explosives, fire or flame, hot vapors or object, machinery, and all transportation). Reviewers also collected variables to confirm acute injury, provider administration of any patient risk assessment, and documented use of a screening or risk assessment tool in the medical record. Other variables included documented suicidal ideation, history of self-harm, history of psychiatric hospitalization, indication of a treatment plan, whether the patient was currently in therapy or took medication for a mental health condition, blood alcohol level, and toxicology or laboratory results. Data on 24 additional circumstances and stressors—such as chronic illnesses, relationship stressors, financial stressors, abuse, history of suicide attempts, and legal/criminal problems—were collected and grouped into those with two or more of these factors and those with fewer than two (see Additional file 1 for the full list).

To prepare for the review, reviewers independently classified 17 standard scenarios that ranged from suicidal ideation to unintentional injury based on two existing suicide classification systems (Crosby et al. 2011). Suicidal ideation was defined as thoughts of wanting to be dead or thoughts of killing oneself (Crosby et al. 2011; Posner et al. 2007). Suicide attempt was defined as “behavior that is self-directed and deliberately results in injury or the potential for injury to oneself. There is evidence, whether implicit or explicit, of suicidal intent” (Crosby et al. 2011). This definition is the same as the definition used by Posner and colleagues: “A potentially self-injurious behavior, associated with at least some intent to die, as a result of the act” (Posner et al. 2007). Reviewers could bring questions to the group of lead study staff from the three states.

### Study size

To determine the desired sample size, the study team used 80% as the expected PPV of intentional self-harm based on 83% PPV from a manual record review of ED visits among adolescents initially identified using ICD-9-CM codes and an administrative data source (Callahan et al. 2013; Swain et al. 2019). Colorado selected a random sample of 260 eligible ED records from 2019 to achieve a margin of error no larger than 4.7% for the 95% confidence interval of the estimated PPV. Maryland selected all eligible records from 2018 and 2019 to reach 234 study records. Massachusetts selected a random sample of 400 eligible ED records from October 1, 2017, through September 30, 2018, to achieve a margin of error no larger than 3.8% for the 95% confidence interval of the estimated PPV. Study records in the results section refer to all records in the review for Maryland and the sampled records in Colorado and Massachusetts (Table 2).

**Table 2:** Eligible study records and random sample: medical record review of intentional self-harm treated in EDs

Characteristics	Maryland	Colorado		Massachusetts	
	% (*N* = 234)	% in frame (*N* = 8551)	% in sample (*n* = 252)^†^	% in frame (*N* = 6651)	% in sample (*n* = 396)^‡^
Intentional self-harm ICD-10-CM codes					
Only specific codes	84.6	91.0	85.3	90.0	90.2
Only nonspecific suicide attempt (T14.91 code)	7.7	2.9	2.8	2.3	2.8
Both specific codes and nonspecific suicide attempt	7.7	6.1	11.9	7.7	7.1
2 or more specific intentional self-harm codes	53.0	19.7	36.9	17.6	16.7
Suicidal ideation (R45.851 code)	35.9	25.0	23.0	22.1	21.0
Mechanism^a^					
Poisoning—Drug	39.5	48.1	54.7	39.9	40.2
Poisoning—Non-drug^b^	8.8	2.2	2.4	4.0	5.1
Cut/pierce	31.6	28.7	22.6	38.6	36.4
Suffocation^c^	8.3	1.7	2.0	1.1	1.0
Other^d^	12.3	16.4	15.5	17.6	18.7
No mechanism^e^	5.7	2.9	2.8	2.3	2.8
Age 18 years and older^a^	69.7	68.8	72.2	75.2	76.0
Female sex	49.6	61.0	56.7	60.9	61.4
Discharged to					
Home	81.2	44.3	38.5	52.2	48.0
Psychiatric unit	18.4	38.9	44.4	N/A	N/A
Other	0.4	16.8	17.1	47.8	52.0

^a^Maryland: *N* = 228 for mechanism, *N* = 231 for age

^b^Toxic effects of non-medicinal substances

^c^Asphyxiation, suffocation, hanging

^d^Includes firearm, fire or flame, hot object or substance, fall, transportation, struck by or against, drowning or submersion, natural or environmental, each of which total less than 1%. Also includes X83.8 for “Intentional self-harm by other specified means”

^e^Colorado: Only nonspecific suicide attempt (T14.91 code)

^†^Colorado: The Colorado Hospital Association selected the sample, of which eight records did not link back to the limited version of the ED billing data set that the health department has access to. These eight records were included in the medical record review

^‡^Massachusetts: Massachusetts inadvertently included four deaths in the original 400 sampled records and did not review their medical records, since deaths were an exclusion criterion for all sites

### Statistical methods

Staff from the three states independently conducted the data analysis for this study using SAS V9.4 and IBM SPSS V23.0. Staff calculated percentages and odds ratios for categorical variables and the mean and standard deviation for age. If the 95% confidence interval for an odds ratio did not include 1, then the odds ratio was considered statistically significant. Positive predictive value was calculated three ways (Table 3) to identify any difference in physician diagnosis, physician documentation, and all documentation in the medical record. For the first PPV calculation, the numerator was limited to records with physician diagnosis of intentional self-harm, based on keywords in the physician’s diagnosis. For the second PPV, the numerator was the number of records with a physician diagnosis or other physician documentation in the medical record that would support an ICD-10-CM code for intentional self-harm. For the third PPV, the numerator was the number of records with any documentation in the medical record indicating intentional self-harm, based on the reviewer’s assessment. The denominator for all three PPV estimates was the total number of sampled records eligible for the review, with eligibility confirmed based on information in the medical record. (Maryland did not sample records but selected all eligible records in the time frame.) The subgroup with a physician diagnosis of intentional self-harm was compared to the subgroup without a diagnosis (Table 4). The subgroup with a physician diagnosis of intentional self-harm was further divided into two groups and compared: those with known or suspected suicidal intent and those with known or suspected non-suicidal intentional self-harm (Table 5).

**Table 3:** Positive predictive value (PPV) of ICD-10-CM codes: Intentional self-harm treated in EDs

	Maryland^†^ *N* = 225		Colorado^†^ *N* = 246		Massachusetts^†^ *N* = 367	
	*n*	%	*n*	%	*n*	%
PPV based on physician diagnosis^a^	202	89.8	226	91.9	357	97.3
PPV based on physician documentation or diagnosis^a^	229	97.9	242	98.4	363	98.9
PPV based on any documentation^a^	229	97.9	243	98.8	363	98.9

^†^Maryland: *N* = 225 (96.2%) of the 234 records eligible for review for the first definition of PPV and 234 (100%) for the second and third definitions of PPV. Colorado: *N* = 246 (94.6%) of the 260 sampled and Massachusetts *N* = 367 (91.8%) of 400 had completed reviews

^a^Reviewers requested and reviewed the full ED report, ambulance/EMT record (EMS transport record), face sheet, and toxicology reports. For all estimates of PPV, the denominator is all reviewed record in the random sample. For the first PPV, the numerator is limited to only records with physician diagnosis of intentional self-harm. For the second PPV, the numerator is the number of records with a physician diagnosis or other physician documentation in the medical record that would support a coder confirming an ICD-10-CM code for intentional self-harm. For the third PPV, the numerator is the number of records with any documentation in the medical record indicating intentional self-harm

**Table 4:** Characteristics associated with predictive value based on physician diagnoses: intentional self-harm treated in EDs

	Maryland *N* = 225		Colorado *N* = 246		Massachusetts *N* = 367	
	Positive (*n* = 202) %	Negative (*n* = 23) %	Positive (*n* = 226) %	Negative (*n* = 20) %	Positive (*N* = 357) %	Negative (*N* ≤ 11) %
**Variables from the medical record**						
Mean age (SD)^a^	28.5 (15.3)	28.6 (14.3)	28.6 (14.4)	28.4 (13.3)	29.5 (14.02)	–
Female^a^	51.5	34.8	58.4	–	61.3	–
Acute injury^b^	71.3	34.8	56.2	–	95.8	–
Drug poisoning mechanism^c^	40.0	43.5	53.1	60.0	43.1	–
Cut/pierce mechanism^c,d^	34.0	13.0	28.8	–	37.5	–
Suicidal ideation	61.4	60.9	80.5	65.0	52.4	–
Risk evaluation and used an assessment tool	63.4	47.8	64.6	65.0	44.1	–
Safety or counseling plan	76.9	70.0	93.4	90.0	81.8	–
Personal history of self-harm	54.0	54.5	56.2	70.0	56.3	–
Prior psychiatric hospitalization^e^	31.8	40.9	27.4	–	34.7	–
Mental health therapy and/or medication	55.9	56.5	61.1	70.0	60.8	–
Blood alcohol 80 + mg/100 ml (among tested)^f^	15.9	21.4	18.0	–	62.7	–
Drugs other than alcohol	39.0	60.9	51.8	–	25.8	–
2 or more personal factors or circumstances	85.1	73.9	86.3	85.0	86.0	–
**Variables from ED billing record**						
Drug poisoning codes^c,g^	n/a	n/a	61.2	72.2	40.6	–
Cut/pierce codes^c,g^	n/a	n/a	26.5	–	36.7	–
Suicidal ideation (R45.851 code)	n/a	n/a	23.0	–	18.8	–
Personal history of self-harm (Z91.5 code)	n/a	n/a	12.8	–	5.0	–
Unspecified suicide attempt (T14.91 code)	n/a	n/a	12.8	–	9.6	–
Not discharged home^h^	n/a	n/a	63.5	–	51.0	–

*%* percentage, *SD* standard deviation, *n/a* not available, —Censored per data use agreement; *CI* confidence interval

^a^Colorado and Massachusetts: information from the ED billing record, not the medical record

^b^Maryland: odds ratio 4.7 (95% CI 1.9–11.6)

^c^If a person attempted a drug overdose and cut/pierce on the same occasion that person is counted twice, once in each category

^d^Maryland: odds ratio 3.4 (95% CI 1.0–12.0)

^e^Maryland: *N* = 22

^f^Colorado: *N* = 196 Maryland: *N* = 14 Massachusetts: *N* = 51

^g^Colorado missing *n* = 32 or 13% of 246

^h^Colorado missing *n* = 4

**Table 5:** Characteristics of physician-diagnosed suicide attempts and non-suicidal intentional self-harm treated in EDs

	Maryland *N* = 202			Colorado^†^ *N* = 226			Massachusetts^†^ *N* = 357		
	Known non-suicidal (*N* = 49) %	Known suicidal (*N* = 131) %	Odds ratios* (95% CI)	Non-suicidal (*N* = 32) %	Suicidal (*N* = 175) %	Odds ratios* (95% CI)	Non-suicidal (*N* = 121) %	Suicidal (*N* = 203) %	Odds ratios* (95% CI)
**Variables from the medical record**									
Mean age (SD)^a^	23.9 (12.9)	31.3 (16.0)		26.0 (12.7)	28.9 (14.7)		26.7 (12.4)	31.1 (14.7)	
Female^a^	55.1	49.6		59.4	60.0		59.5	66.0	
Acute injury	77.6	73.3		62.5	54.3		97.5	94.6	
Drug poisoning mechanism^b^	16.3 (8)	49.6	5.0 (2.2–11.6)	34.4	57.7	2.6 (1.1–6.3)	7.4	65.0	23.1 (11.1–48.4)
Cut/pierce mechanism^b^	59.2	24.4	0.2 (0.1–0.5)	53.1	26.3	0.31 (0.14–0.73)	72.7	16.7	.08 (0.04–0.13)
Suicidal ideation	24.5	77.1	10.4 (4.8–22.4)	50.0	92.6	12.5 (4.6–33.4)	32.2	69.5	4.8 (2.9–7.8)
Risk evaluation and used an assessment tool	59.2	63.4		65.6	65.1		39.2	47.5	
Safety or counseling plan	71.4	79.8		93.8	94.3		77.7	89.2	
Personal history of self-harm	46.9	59.5		56.3	55.4		66.9	54.2	0.58 (0.37–0.93)
Prior psychiatric hospitalization	32.7	33.1		–	27.4		29.8	38.9	
Mental health therapy and/or medication	59.2	57.3		53.1	62.9		64.5	61.6	
Blood alcohol 80 + mg/100 ml^†^	7.1 (3)	22.1	3.7 (1.01–13.4)	–	16.3		–	12.3	4.1 (1.4–12.0)
Drugs other than alcohol	33.3	42.3		–	56.6		18.2	31.0	
2 or more personal factors or circumstances	79.6	90.8	2.5 (1.02–6.3)	81.3	88.6		83.5	90.1	
**Variables from ED billing record**									
Drug poisoning codes^b,c^	n/a	n/a		37.5	69.2	3.7 (1.6–9.1)	–	61.6	26.1 (11.6–58.9)
Cut/pierce codes^b,c^	n/a	n/a		50.0	21.2	0.3 (0.1–0.7)	70.2	17.2	.09 (.05–.15)
Suicidal ideation (R45.851 code)	n/a	n/a		–	24.5		21.5	18.2	
Personal history of self-harm (Z91.5 code)	n/a	n/a		–	13.1		6.6	4.9	
Unspecified suicide attempt (T14.91 code)	n/a	n/a		–	15.4		–	15.3	7.1 (2.1–23.7)
Not discharged home^d^	n/a	n/a		–	74.9	7.6 (3.1–20.0)	31.4	66.5	4.3 (2.7– 7.0)

*%* percentage, *CI* confidence interval, *SD* standard deviation, *n/a* not available, —Censored per data use agreement

^†^CO: Known intentional self-harm of whom 28 are suspected suicidal (16% of 175) and 25 suspected of non-suicidal intentional self-harm (78% of 32). MA: Known intentional self-harm with suspected or known combined for each type (suicidal or not)

^*^Shown are odds ratios that do not include 1. The referent group was the non-suicidal intentional self-harm group, based on physician diagnosis

^a^Colorado and Massachusetts: information from the ED billing record, not the medical record

^b^If a person attempted a drug overdose and cut/pierce on the same occasion that person is counted twice, once in each category

^c^CO missing *n* = 29 (14%) of the 207

^d^CO missing *n* = 4 of 207

## Results

Of the 234 study records from the Maryland study site, 225 or 96.2% were eligible for review, based on information in the electronic medical record. Similarly, of the 260 study records from Colorado, 246 or 94.6% were eligible for review based on information from the medical record. Most ineligible records in Colorado were ineligible because the patient was admitted as an inpatient. Of the 400 study records from Massachusetts, 367 or 91.8% were available and eligible for review. Twenty-eight (7%) of Massachusetts records were not received in time for the review.

According to the billing database for emergency department visits, the percentage of study records with only specific ICD-10-CM codes ranged from 85 to 90% across the study sites (Table 2). Almost 3% of the eligible study samples in Colorado and Massachusetts and almost 8% in the Maryland site had the ICD-10-CM code T14.91 for nonspecific suicide attempt. Twenty-one percent to 36% of the study records from the billing database included the ICD-10-CM code for suicidal ideation. Drug poisoning was the most common mechanism specified (40–55%), followed by cutting or piercing (23–36%). More than two-thirds were adults 18 years or older. Half to 61% were female. More patients in Colorado (44%) were coded as having been discharged to a psychiatric unit, compared to Maryland (18%). The Massachusetts billing database and review did not document transfers to a psychiatric unit or facility.

The estimated positive predictive value of the ICD-10-CM codes for intentional self-harm based on documented physician diagnosis is shown in Table 3. PPV was 89.8% for Maryland study records (95% confidence interval 85.0–93.4), 91.9% for Colorado study records (95% confidence interval 87.7–95.0), and 97.3% for Massachusetts (95% confidence interval 95.1–98.7). When all physician documentation in the medical record was consulted, the positive predictive value became similar across the study sites: 97.9% in Maryland, 98.4% in Colorado, and 98.9% in Massachusetts. The PPV estimate for Maryland and Massachusetts did not change, and the Colorado PPV increased only 0.4% when the reviewers assessed all documentation in the medical record.

Few study records with an ICD-10-CM billing code for intentional self-harm lacked physician diagnosis of intentional self-harm documented in the medical record (shown in the column labeled “Negative” in Table 4). Therefore, it was difficult to detect statistically significant differences between characteristics associated with positive or negative findings. The few exceptions were from the Maryland study. Seventy-one percent of the Maryland study records with a documented physician diagnosis of intentional self-harm had an acute injury, compared to only 34.8% of the patients without documented physician diagnosis (odds ratio 4.7, 95% confidence interval 1.9–11.6). Thirty-four percent of the Maryland study records with a documented physician diagnosis of intentional self-harm had cut or pierce as the specific mechanism of injury, compared to 13% of records without such intentional self-harm documentation (odds ratio 3.4, 95% confidence interval 1.0–12.0). In general, compared to the records with a physician diagnosis of intentional self-harm in the medical record, few records without a physician diagnosis had a similar proportion of the characteristics of interest.

The type of documented intentional self-harm varied by study site. In Maryland, 64.9% of the study records with documented physician diagnosis were suicidal, compared to 77.4% in Colorado and 56.9% in Massachusetts. When comparing suicidal and non-suicidal intentional self-harm among the study records with documented physician diagnosis of intentional self-harm, the mean age was slightly higher for the suicidal group in each site (Table 5). The majority of both groups in all three sites were female (ranging from 49.6 to 66.0%). Factors significantly associated with the suicidal group in all three sites were drug poisoning and suicidal ideation. In Maryland and Massachusetts, a blood alcohol concentration of 80 or more mg per 100 ml was associated with suicidal self-harm. Cutting or piercing as a mechanism of intentional self-harm was associated with the non-suicidal group. In Colorado and Massachusetts, the odds ratios for drug poisoning and cut/pierce were similar whether calculated using information from the source medical record or the billing record. In Colorado, patients in the suicidal group were 7.6 times more likely not to be discharged home than patients in the group with non-suicidal intentional self-harm. In Massachusetts, the suicidal group was 4.3 times more likely not to be discharged home.

## Discussion

This retrospective medical record review estimated that the ICD-10-CM codes for intentional self-harm in ED billing records for patients ages 10 years and older had a 90% or greater positive predictive value, based on documented physician diagnosis of intentional self-harm in the original medical record. This finding, the main study objective, was similar to the finding of 88% PPV for the ability of ICD-10-CM codes to capture intentional self-harm injuries among patients under 18 years of age by Hansen et al. (2021). Our PPV finding for ED visits among patients ages 10 years and older was slightly higher than the 83% PPV of ICD-9-CM codes to capture ED visits for suicidal behavior among adolescents found by Callahan et al. (2013) and Swain et al. (2019). The similar magnitude of our PPV finding with the PPV from these adolescent studies might reflect that a large proportion of our study population were children and adolescents. Specifically, about 24–30% of our study population were aged 10–17 years old. The authors did not detect variables in the ED billing data set associated with the few study records that did not have a documented physician diagnosis of intentional self-harm; the purpose of such variables would be to refine the estimate of non-fatal intentional self-harm when analyzing ED billing records alone. The high PPV findings are important to epidemiologists conducting public health surveillance and researchers selecting intentional self-harm for clinical research, using administrative billing data for ED visits.

Regarding the second study objective, only a few characteristics (drug poisoning, cut or pierce mechanism of injury, and suicidal ideation) were associated with suicide attempts, compared to non-suicidal intentional self-harm, among the records with documented physician diagnosis of intentional self-harm. However, both drug poisoning (7.4–34.4%) and suicidal ideation (24.5–50.0%) were also associated with non-suicidal intentional self-harm, and the confidence intervals for the odds ratios were wide. The records with documented physician diagnoses for suicidal and non-suicidal intentional self-harm had similar results for other characteristics related to patient history, risk evaluation, safety or counseling plan, and mental health therapy and/or medication for a mental health condition. These similarities may reflect the continuum of suicidality and the consistent standard of care in the ED for patients presenting with suspected or known intentional self-harm. However, these factors may also be present in both types of self-harm because non-suicidal intentional self-harm can be a risk factor for future suicide attempts (Ribeiro et al. 2016). This inability to definitely distinguish suicide attempts from non-suicidal intentional self-harm would limit the usefulness of ICD-10-CM billing data for surveillance of suicide attempts or for the starting point of research about suicide attempts.

Though not an objective of this study, this study did describe the use of the ICD-10-CM code T14.91 for “suicide attempt.” This nonspecific code was the only intentional self-harm code for less than 3% of the eligible records for sampling (sampling frame) and study sample in Colorado and Massachusetts and almost 8% in the Maryland hospital ED. The low percentages in the two states might indicate quality, comprehensive documentation, and coding in those states. About 6–8% of the eligible records for sampling (the sampling frame) in the two states and the Maryland ED had both an intentional self-harm code with a mechanism specified and the nonspecific suicide attempt code T14.91, which was not in accordance with the ICD-10-CM guidance. Beyond the scope of this study, a future study could determine the reason(s) for the use of a specific intentional self-harm code with code T14.91, such as to indicate that the self-harm was suicidal.

### Limitations

This study did not assess sensitivity and specificity and therefore does not address the magnitude of the intentional self-harm that these ICD-10-CM codes capture or miss. Our data are limited to EDs affiliated with nonfederal acute care hospitals in three states in the USA and may not be generalizable to other states or health care settings, such as an inpatient medical service at a hospital, or to people who died. The results from the single Maryland hospital ED may not be generalizable to other Maryland EDs. Four to eight percent of the study records across the three states were not reviewed, either because the medical records were unavailable or because they did not meet the eligibility criteria for the study. If these records were counted in the denominator for the PPV calculation, the PPV estimates would be lower.

Heterogeneity presented a challenge. Anecdotally, eligible study records in Colorado included self-harm behavior that was not close in time to the ED visit, episodes when the behavior was aborted (such as a patient spitting out pills), and instances when the patient was having a psychotic or manic episode. The implications of this type of heterogeneity for the study findings remain unclear. Another limitation arises from reliance on documentation in the medical record. The use of electronic health record systems in hospitals does not necessarily eliminate variation in documentation (Glynn and Hoffman 2019). Furthermore, in Colorado and Massachusetts, the reviewers did not always receive all the documents requested. Colorado hospitals provided the key documents from the medical record: the physician notes, psychiatric consultation, and EMS reports for almost all study records and also history and physical, triage notes, and laboratory reports for most study records. In Colorado and Massachusetts, the reviewers were professional medical coders, not clinical staff, which could introduce variability in interpretation of physician documentation. However, the Colorado and Massachusetts results were not consistently different from the Maryland results collected by clinical researchers.

The study identified a group of records with documented physician diagnosis of intentional self-harm for which the reviewer was unable to distinguish between suicidal and non-suicidal intent because of conflicting information or lack of documentation about intent to die. This group of records represented 10.9% of documented intentional self-harm records in Maryland, 8.4% in Colorado, and 9.2% in Massachusetts. This group of records with unknown intent to die illustrates the challenges in diagnosing suicidal and non-suicidal self-harm (Hooley et al. 2020).

Further studies are needed to identify sensitivity and specificity of the ICD-10-CM codes for intentional self-harm. Such sensitivity and specificity studies could sample from billing records with a only suicidal ideation code (R45.851) without any intentional self-harm codes and from billing records with only non-suicidal self-harm (R45.88, a new ICD-10-CM code implemented on October 1, 2021) without any intentional self-harm code. Future studies are needed to determine ways to distinguish between suicide attempts and non-suicidal intentional self-harm using ICD-10-CM coded data set. These studies could improve upon this study by increasing the sample size to increase the power to detect a difference between suicidal and non-suicidal self-harm. Also, a future study could focus on the subgroup that had more than one specific ICD-10-CM code for intentional self-harm, given that 17–53% of the population from which this study was drawn had multiple self-harm codes.

## Conclusions

Overall, a multi-site medical record review of ED visits among patients ages 10 and older identified in administrative billing databases with ICD-10-CM codes for intentional self-harm found high positive predictive value (90% or greater). Therefore, the ICD-10-CM codes in question meet the threshold for use in public health surveillance of intentional self-harm. However, these codes and additional variables in the billing databases examined in this study do not distinguish suicidal from non-suicidal intentional self-harm, critical both for surveillance and clinical research. A potential solution would require a change in ICD-10-CM guidance allowing professional coders to code the symptoms of suicidal ideation (R45.851) and non-suicidal self-harm (R45.88) in addition to the diagnosis of intentional self-harm.

## Supplementary Information

**Additional file 1**. List of personal factors or circumstances. Medical record reviewers collected this list of personal factors or circumstances documented in the medical records of intentional self-harm treated in the emergency departments in the study sites of Colorado, Maryland, and Massachusetts.

## Data Availability

Data are not available because of data use agreement restrictions.

## Abbreviations

CI: Confidence interval
ED: Emergency department
ICD-10CM: International Classification of Disease, Tenth Revision, Clinical Modification used in the USA
PPV: Positive predictive value
