# Supplementary material for: Multi-site medical record review for validation of intentional self-harm coding in emergency departments
Source: Inj Epidemiol. 2022 Jun 7;9:16. doi: 10.1186/s40621-022-00380-y (PMC9175468; doi:10.1186/s40621-022-00380-y)
Supplement: Supplementary file 1 — Additional file 1. List of personal factors or circumstances. Medical record reviewers collected this list of personal factors or circumstances documented in the medical records of intentional self-harm treated in the emergency departments in the study sites of Colorado, Maryland, and Massachusetts. [file 40621_2022_380_MOESM1_ESM.docx]

**Supplemental File: List of personal factors or circumstances**

This list was adapted from two sources:

Crosby AE, Ortega L, Melanson C. Self-directed Violence Surveillance: Uniform Definitions and Recommended Data Elements, Version 1.0. Atlanta (GA): Centers for Disease Control and Prevention, National Center for Injury Prevention and Control; 2011. <https://www.cdc.gov/suicide/pdf/Self-Directed-Violence-a.pdf>

The U.S. Centers for Disease Control and Prevention: The National Violent Death Reporting System. <https://www.cdc.gov/violenceprevention/datasources/nvdrs/> Accessed 09 Nov 2021.

The instruction to the medical records reviewer: Check off all circumstances documented below as positive in the ED record. Any documentation in the ED record is valid, regardless of the informant.

- History of depressed mood disorder
- History of other mental health disorder
- History of mental health treatment (meds/therapy)
- History of substance abuse/dependence disorder
- History of substance abuse/dependence treatment
- History of mental illness in family
- Caregiver stressors (caregiving for family member) – Maryland did not collect.
- Patient suffers from chronic/multiple illnesses
- Crisis within last two weeks
- Work- or school-related stressors
- Relationship stressors
- Financial stressors
- Victim of bullying
- Victim of abuse
- History of interpersonal violence
- Real or perceived discrimination
- Has physical disability or health problem
- Recent death of friend/family, not necessarily self-harm
- Anniversary of a traumatic event
- Eviction or loss of home
- History of suicide attempts
- History of expressed suicidal thoughts or plans
- Legal/criminal problems
- Current/recent prisoner
